# Inequalities in Structural Social Capital and Health between Migrant and Local Hypertensive Patients

**DOI:** 10.5334/aogh.2398

**Published:** 2019-03-27

**Authors:** Wu Zhu, Haitao Li, Hui Xia, Xuejun Wang, Chen Mao

**Affiliations:** 1 School of Management, Wuhan University, Wuhan, CN; 2 Shenzhen University General Hospital, Shenzhen University Academy of Clinical Medical Sciences, Shenzhen, CN; 3 Center for Chronic Disease Prevention and Control, Shenzhen, CN; 4 School of Public Health, Southern Medical University, Guangzhou, CN

## Abstract

**Background:**

Inequality in health between migrant and local hypertensive patients is an important public health concern. This study aims to examine the associations of registration status with structural social capital and health of hypertensive patients, as well as how structural social capital operates in the relationship between registration status and health.

**Methods:**

We conducted an on-site based cross-sectional study in Shenzhen, China. A total of 1046 participants completed the survey. Information with respect to structural social capital, subjective and objective health outcomes was collected. Multiple logistic or linear regression models were used to test the associations across registration status, structural social capital and health outcomes.

**Results:**

The findings show that migrant hypertensive patients have lower structural social capital in terms of social contacts (10.87 vs. 10.41; β = –0.457, 95% CI: –0.866, –0.048) and poorer health outcomes, i.e., blood pressure control (56.4 vs. 43.6%; OR = 0.557, 95% CI: 0.364, 0.852) when compared to the local individuals. Meanwhile, individuals with lower structural social capital report poorer self-rated health. However, the differences in structural social capital between migrant and local hypertensives explain only a small proportion of the large inequalities in blood pressure control.

**Conclusions:**

Inequalities exist between migrant and local hypertensives in objective but not subjective health outcomes. Structural social capital associates with subjective health outcomes of hypertensive patients only. A modest extent of medication is found by structural social capital of registration health inequalities. Our study suggests that growing contacts providing support for migrant hypertensive patients should be an important goal of future strategies and policies. It also highlights the need for more research on mediating and moderating mechanisms in order to understand the relationship between registration status and health outcomes among hypertensive patients.

## Introduction

Hypertension is a major public health problem faced by policy makers worldwide and China. It has been projected that, in 2025, there will be 1.56 billion adults living with hypertension globally [1]. This figure will significantly increase if the new definition of hypertension applied i.e., SBP ≥ 130 mmHg or DBP ≥ 80 mmHg [2]. Besides its high prevalence, hypertension is one of the major risk factors for cardiovascular and cerebrovascular diseases. Awareness, treatment and control of hypertension still remain suboptimal [3,4], representing one of the largest health challenges and high cost disease for individuals and society.

Social inequalities are an important public health topic concerning the entire population. Literature shows that migration is correlated with increased risks in mental health and health behaviors [5,6]. As far as hypertension is concerned, a large body of international and national evidence documents the association of registration status and health across hypertensive patients. The migrant hypertensive patients usually have lower rates of awareness [7], treatment [8] and control [9] in comparison to their local counterparts. The inequalities in health between migrants and the locals may affect the “health for all” target. Social capital has been incorporated in the WHO’s general conceptual framework on the social determinants of health [10]. Social capital has been shown to play an important mediating [11] or moderating [12] role in decreasing social inequalities in health. However, studies investigating the mechanisms through which social capital operates among hypertensive patients are scarce.

Social capital refers to features of social organization, such as networks, norms and social trust that facilitates coordination and cooperation for mutual benefit [13]. Conceptualization of social capital can be decomposed into a structural and cognitive component [14]. Structural social capital is defined as the objectively measurable characteristics such as participation in clubs, neighborhood activities and other social networks. Portes [15] has described social capital as an individual attribute and Kawachi et al. [16] highlight the question of the individual/collective distinction of social capital. There is increasing evidence that individual-level structural social capital is associated with better health. The study by Eriksson et al. [17] showed a positive association between individual structural social capital and self-rated health. However, current studies are more on an association of individual structural social capital with subjective health, leaving the association with objective health outcomes tenuous.

Shenzhen is one of the most populous metropolitan areas located in the Pearl River Delta region of southern China. It is China’s first Special Economic Zone holding sub-provincial administrative status. It is divided into 10 district-level jurisdictions. The total area of Shenzhen is 1997.3 square kilometers. According to the statistics for 2016, Shenzhen’s population is around 11.4 million with about 69% being migrants [18]. Migrants here refers to internal migrants who do not change their official *Hukou* registration to the new location to which they move (i.e., floating or non-permanent residents) [19]. *Hukou* refers to a household registration status officially issued, often on a family basis, to identify a person’s official place of residence [20]. *Hukou* defines a person’s access to employment, housing, social welfare, education opportunities and medical and other services. Migrants in Shenzhen normally move from rural areas for better paid jobs. They are generally less skilled and minimally educated and, therefore, tend to have lower incomes than their local counterparts. The prevalence of hypertension in Shenzhen has been increasing in the past decade, measured at about 20% in 2016. Shenzhen offers an interesting case study to examine the effect of structural social capital on the relationship between social inequalities and health among hypertensive patients.

This study aims to investigate the association of registration status with health outcomes of hypertensive patients and how structural social capital operates this relationship. The following sets of hypotheses will be tested:

1. Migrant status has a direct negative effect on structural social capital, as well as health outcomes of hypertensive patients.
2. Individual-level structural social capital associates with both subjective and objective health outcomes of hypertensive patients.
3. Individual-level structural social capital mediates the relationship between registration status and health outcomes of hypertensive patients.

## Methods

### Sample and procedures

This was a cross-sectional study conducted in Shenzhen, China. Community health centers (i.e., primary care facilities) were selected as study settings by using multistage cluster random sampling methods. Initially, one of the ten districts in Shenzhen was selected employing simple random sampling methods, i.e., Longhua District (including six sub-districts). In the second stage, the list of community health centers for each sub-district was obtained from Health Bureau of Longhua District. Two community health centers were randomly drawn from each sub-district employing a simply random sampling method. Finally, a total of twelve community health centers were selected.

The survey was on-site based. The sampling frame was primary care users’ population based. Employing a systematic sampling design, every 5^th^ care user was selected. The inclusion criteria included, (1) aged ≥18-year old; (2) ability to communicate and give informed consent; and (3) had been living in Shenzhen for more than six months. The selected primary care users were asked whether they had previously been diagnosed with hypertension by health care professionals. A measurement of blood pressure was also administered. The selected respondents should either have (1) hypertension diagnosed by health care professionals; or (2) an elevated blood pressure (≥140/90 mmHg) measurement. Hypertensive patients were consecutively approached until the first one hundred had been recruited for each community health center (1200 in total). Extensively trained interviewers performed face-to-face interview surveys between March and September 2017. The respondents were assured of the anonymity and confidentiality of the survey, and informed consent was obtained before the surveys commenced. In sum, 1046 respondents completed the survey with the response rate of 87.2%.

### Variables

Health outcomes were measured both objectively and subjectively. Hypertension control was defined as achieving and maintaining blood pressure levels below 140 and 90 mmHg for SBP and DBP respectively. The respondents were then dichotomized into two groups, i.e., the optimal (SBP < 140 mmHg and DBP < 90 mmHg) and the suboptimal (SBP > 140 mmHg or DBP > 90 mmHg). Self-reported health was assessed by asking the respondents if they, generally speaking, would say that their current health was excellent, very good, good, fair, or poor. For analysis purposes, we dichotomized respondent responses into high (excellent, very good and good) and low (fair and poor) categories.

Structural social capital was measured at the individual level using the decomposition of participation in organizations (formal networks potentially bridging) and social contacts (informal networks indicative of bonding ties). For the measurement of participation in organizations/groups, the respondents were asked: Are you involved in the any of the following kinds of organizations? Response categories were political parties, sports teams, associations like technology, religious organizations, volunteers, same hobby teams, colleagues, family, fellow-townsman, classmates (0 = no, 1 = yes). An unweighted sum score was calculated (range 0–10). Social contacts were measured by three items, (1) How often are you in contact with your family members or relatives? (2) How often are you in contact with your friends? (3) How often are you in contact with your neighbors? Responses included: 1 = never; 2 = seldom; 3 = sometimes; 4 = frequently; 5 = always. An unweighted sum score ranging between 3 and 15 was calculated.

Covariates included age in years (1 = 18–44 years; 2 = 45–6 years; 3 = >60 years), gender (1 = male; 2 = female), education level (1 = primary school and below; 2 = middle school; 3 = high school or equivalent; 4 = three-year college and above), occupation (1 = employed; 2 = unemployed), year of hypertension since being diagnosed, family history of hypertension (1 = yes; 2 = no; 3 = unknown).

### Statistical analyses

We used SPSS 20.0 to conduct all analyses. Descriptive statistics of migrant and local respondents were calculated in terms of age, gender, education, occupation, year of hypertension, family history of hypertension, structural social capital and health outcomes. A comparison of social-demographic factors, structural social capital and health outcomes, between migrant and local respondents, was performed using Chi-square test (or t-test where appropriate). Multiple linear regression models were used to test the associations between registration status and structural social capital. Multiple logistic regression models were constructed to test the associations between registration status and health outcomes and, across registration status, structural social capital and health outcomes. The latter decomposed the effect estimates into direct and indirect effects. All models were adjusted for age, gender, education, occupation, year of hypertension and family history of hypertension. Odds ratios or βs (95% confidence intervals) were reported. All *P*-values <0.05 were considered as statistically significant.

## Results

Of all respondents, mean age was 55.48 years, predominantly male (59.8%) and employed (55.0%). Mean duration of hypertension was 6.08 years. Among the 1046 respondents, 216 were locals and 830 were migrants. Compared with the locals, migrants were more likely to be younger (P < 0.001), have a lower education level (P < 0.001), and be employed (P = 0.002). Mean years of having a hypertension diagnosis was 7.27 and 5.82 for the locals and migrants, respectively (P = 0.009). More local respondents did not have a family history of hypertension compared to their migrant counterparts (P = 0.046) (Table 1).

**Table 1:** Socioeconomic characteristics of the migrant and local hypertensive patients.

Characteristics	Total		Locals		Migrants		*P*

	Frequency	Percentage (%)	Frequency	Percentage (%)	Frequency	Percentage (%)	

Age (n = 996) (mean, SD)	55.48	11.35	59.40	11.96	54.82	11.15	<0.001
18–44	172	17.3	15	11.2	149	18.7	0.001
45–60	476	47.8	53	39.6	389	48.7	
>60	348	34.9	66	49.3	260	32.6	
Gender (n = 977)							
Male	584	59.8	77	58.3	468	59.7	0.768
Female	393	40.2	55	41.7	316	40.3	
Education (n = 1027)							
Primary school and below	283	27.6	32	23.0	231	28.4	<0.001
Middle school	359	35.0	37	26.6	296	36.4	
High school and equivalent	262	25.5	37	26.6	209	25.7	
3-year college and above	123	12.0	33	23.7	78	9.6	
Occupation (n = 1020)							
Employed	561	55.0	60	42.6	460	56.6	0.002
Unemployed	459	45.0	81	57.4	353	43.4	
Year of hypertension (mean, SD)	6.08	6.00	7.27	6.41	5.82	5.70	0.009
Family history of hypertension (n = 996)							
Yes	454	45.6	65	48.1	359	45.3	0.046
No	377	37.9	57	42.2	290	36.6	
Unknown	165	16.6	13	9.6	144	18.2	

Using Chi-square tests, we compared individual structural social capital and health outcomes across the respondents with different socio-demographic characteristics (Table 2). The locals had a higher intensity of social contact when compared to their migrant counterparts (10.87 vs. 10.41, P < 0.05). Intensity of social contacts declined with age (P < 0.05). The employed participated more in organizations (2.38 vs. 1.75, P < 0.001), and had a higher intensity of social contact (10.62 vs. 10.32, P < 0.05) compared with the unemployed. In general, the number of organizations (P < 0,001) and the intensity of social contacts (P < 0,001) increased with the level of education. Male respondents were more likely to participate organizations (P < 0.05). Hypertension control rate was higher in the locals than that in the migrants (56.4 vs. 43.6%, P < 0.01), while no statistically significant difference was observed for self-reported health.

**Table 2:** Social capital and health outcomes of the respondents by sociodemographic characteristics.

Characteristics	Social capital, mean(SE)				Health outcomes, n(%)			

	Organizations	*P*	Contacts	*P*	BP control	*P*	SRH	*P*

***Registration status***								
Locals	2.33(0.19)		10.87(0.17)	*	79(56.4)	**	99(73.9)	
Migrants	2.06(0.07)		10.41(0.07)		360(43.6)		525(68.4)	
***Covariates***								
Age (n = 996)								
18–44	2.30(0.15)		10.74(0.16)	*	68(39.8)		106(66.3)	
45–60	2.10(0.10)		10.57(0.09)		222(47.2)		311(69.7)	
>60	1.93(0.12)		10.24(0.11)		158(45.9)		222(68.7)	
Gender (n = 977)								
Male	2.22(0.09)	*	10.47(0.09)		263(45.7)		374(68.6)	
Female	1.86(0.11)		10.46(0.10)		182(46.7)		252(69.6)	
Education (n = 1027)								
Primary school and below	1.38(0.10)	***	10.10(0.12)	***	118(42.0)		178(67.4)	
Middle school	1.97(0.11)		10.60(0.11)		172(48.5)		229(70.0)	
High school and equivalent	2.64(0.14)		10.84(0.12)		119(45.6)		170(70.0)	
3-year college and above	2.91(0.21)		10.42(0.20)		57(46.3)		82(68.9)	
Occupation (n = 1020)								
Employed	2.38(0.09)	***	10.62(0.09)	*	262(47.3)		358(69.0)	
Unemployed	1.75(0.10)		10.32(0.09)		197(43.3)		296(68.8)	
Family history of hypertension (n = 996)								
Yes	2.12(0.10)	**	10.50(0.10)		197(43.8)		271(63.5)	**
No	2.26(0.11)		10.58(0.11)		184(49.2)		249(71.6)	
Unknown	1.55(0.16)		10.18(0.14)		75(46.3)		119(77.3)	

* *P* < 0.05, ** *P* < 0.01, *** *P* < 0.001.

**Figure 1:**
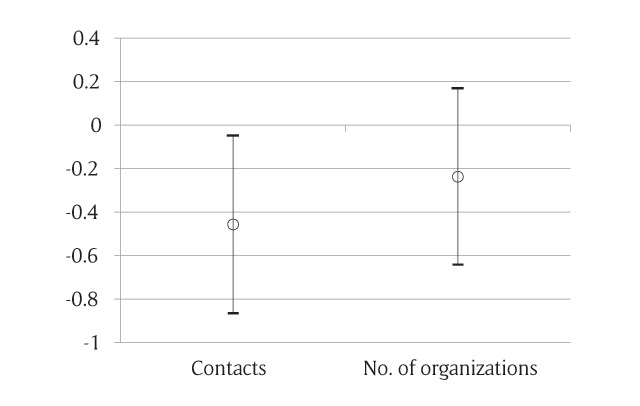
Multiple linear regression models for testing the association between regression status and structural social capital. *Contacts*: –0.457(–0.866, –0.048) P = 0.028. *No. of organizations*: –0.237(–0.642,0.169) P = 0.252.

Using multiple linear regression models, we tested the associations between registration status and structural social capital after controlling for the confounders (Figure 1). A smaller proportion of migrant hypertensive patients belonged to organizations/groups compared to their local counterparts, however, the difference was not statistically significant (β = –0.237; 95% CI: –0.642, 0.169). As for social contacts, after adjustments were made for socio-demographic factors, statistically significant difference still remained (β = –0.457; 95% CI: –0.866, –0.048).

Multiple logistic regression models showed that there was still a statistically significant difference in the hypertension control rate between migrant and local hypertensive patients after adjustment for sociodemographic factors (OR = 0.557; 95% CI: 0.364, 0.852) (Table 3, **model 1**).

**Table 3:** The association between registration status and BP control.

Independent variables	BP control#			

	Model 1	Model 2	Model 3	Model 4

***Registration status***				

Locals	1	1	1	1
Migrants	0.557(0.364,0.852)**	0.576(0.366,0.907)*	0.587(0.382,0900)*	0.602(0.381,0.950)*
***Socio-demographic factors***				

Age (n = 996) (mean, SD)	1.002(0.985,1.019)	1.006(0.988,1.024)	1.002(0.985,1.019)	1.006(0.988,1.024)
Gender (n = 977)				
Male	1	1	1	1
Female	1.341(0.964,1.865)	1.465(1.033,2.079)*	1.300(0.933,1.811)	1.430(1.006,2.033)*
Education (n = 1027)				
Primary school and below	1	1	1	1
Middle school	1.419(0.957,2.102)	1.523(1.002,2.314)*	1.398(0.941,2.077)	1.506(0.88,2.295)
High school and equivalent	1.234(0.801,1.901)	1.194(0.743,1.919)	1.233(0.799,1.904)	1.201(0.747,1.932)
3-year college and above	1.413(0.807,2.472)	1.561(0.860,2.831)	1.411(0.807,2.468)	1.553(0.856,2.819)
Occupation (n = 1020)				
Employed	1	1	1	1
Unemployed	0.814(0.548,1.208)	0.753(0.492,1.153)	0.827(0.556,1.230)	0.758(0.494,1.162)
Year of hypertension	0.985(0.958,1.012)	0.986(0.958,1.014)	0.985(0.959,1.013)	0.986(0.958,1.015)
Family history of hypertension (n = 996)				
Yes	1	1	1	1
No	1.089(0.791,1.500)	1.135(0.809,1.593)	1.077(0.781,1.484)	1.127(0.802,1.584)
Unknown	1.095(0.720,1.663)	1.188(0.756,1.866)	1.119(0.734,1.704)	1.216(0.772,1.915)
***Structural social capital***				

Organizations		0.977(0.900,1.061)		0.975(0.896,1.060)
Contacts			1.012(0.941,1.088)	0.990(0.914,1.072)

# Multiple logistic regression models. * *P* < 0.05, ** *P* < 0.01.

When adding structural social capital variables to multiple logistic regression models (either separately or all domains combined), the estimates of association between registration status and hypertension control attenuated slightly (Table 3, **models 2–4**). In the final model adjusted for sociodemographic factors and all measures of structural social capital, the odds of good health were almost 40% lower for migrant hypertensives compared with the local ones (OR = 0.602; 95% CI: 0.381, 0.950) (Table 3, **model 4**). No statistically significant differences were observed for the relationship between registration status and SRH by using multiple logistic regression models for controlling for confounders (Table 4).

**Table 4:** The association between registration status and SRH.

Independent variables	SRH#			

	Model 1	Model 2	Model 3	Model 4

**Registration status**				

Locals	1	1	1	1
Migrants	1.374(0.849,2.225)	1.408(0.837,2.367)	1.257(0.771,2.049)	1.336(0.789,2.261)
**Socio-demographic factors**				

Age (n = 996) (mean, SD)	0.997(0.979,1.016)	1.003(0.983,1.023)	0.996(0.977,1.015)	1.001(0.981,1.021)
Gender (n = 977)				
Male	1	1	1	1
Female	0.866(0.601,1.247)	0.892(0.605,1.317)	0.907(0.626,1.314)	0.928(0.625,1.377)
Education (n = 1027)				
Primary school and below	1	1	1	1
Middle school	0.814(0.528,1.255)	0.793(0.499,1.261)	0.831(0.535,1.289)	0.785(0.490,1.258)
High school and equivalent	0.805(0.502,1.289)	0.803(0.478,1.348)	0.854(0.529,1.377)	0.835(0.495,1.408)
3-year college and above	0.862(0.470,1.579)	0.855(0.446,1.639)	0.827(0.448,1.524)	0.798(0.413,1.541)
Occupation (n = 1020)				
Employed	1	1	1	1
Unemployed	0.932(0.603,1.440)	0.769(0.481,1.231)	0.888(0.571,1.382)	0.745(0.463,1.200)
Year of hypertension	1.026(0.997,1.056)	1.025(0.995,1.057)	1.024(0.995,1.055)	1.023(0.992,1.055)
Family history of hypertension (n = 996)				
Yes	1	1	1	1
No	0.749(0.528,1.062)	0.676(0.465,0.983)*	0.762(0.535,1.085)	0.686(0.470,1.002)
Unknown	0.556(0.342,0.903)*	0.493(0.291,0.838)**	0.539(0.330,0.879)*	0.487(0.285,0.832)**
**Structural social capital**				

Organizations		0.953(0.868,1.047)		0.983(0.893,1.082)
Contacts			0.870(0.803,0.943)**	0.861(0.788,0.941)**

# Multiple logistic regression models. * *P* < 0.05, ** *P* < 0.01.

## Discussion

Our study examined the relationship between registration status and structural social capital, as well as health outcomes among hypertensive patients. It also investigated the effect of structural social capital on the relationship between registration status and health outcomes. Our study suggested that migrant hypertensive patients had lower structural social capital in terms of social contacts and poorer health outcomes, i.e., BP control when compared to the local individuals. Meanwhile, individuals with lower structural social capital reported poorer SRH. However, the differences in structural social capital between migrant and local hypertensives explained only a small proportion of the large inequalities in BP control.

Results showed that migrant hypertensive patients had lower structural social capital and poorer control of BP when compared to locals, which is consistent with previous findings. A large body of evidence has documented that subjects with lower socioeconomic status have lower social capital [21,22], as well as poorer awareness, treatment and control of hypertension [23,24]. However, the relationship between migrant status and SRH was not identified by the current study and needs further investigation.

Results showed individuals with lower structural social capital reported poorer SRH which echoes previous study findings. Previous individual-level studies of social capital have examined the association of individual social capital with a range of health status or health behavior outcomes. Extensive research has shown associations at the individual level between cognitive social capital including trust [25], social participation [26] and cohesion [27] and SRH. As far as structural social capital is concerned, the study by Mithen et al. [28] found that people with fewer social contacts usually report poorer SRH. The study by Postmes and Branscombe [29] shows that blacks who have more long-term contact with other blacks have also been found to have greater subjective well-being. The migrants usually have smaller networks which may limit the quantity of information available to them. Fewer contacts with family members, friends and neighbors may limit not only the quantity but also the quality of information. However, our study did not observe statistically significant differences between structural social capital and objectively measured health outcomes, i.e. BP control. The association between SRH and self-reported structural social capital may result from a common source bias. Therefore, caution should be made when interpreting the study findings.

The modest extent of medication by structural social capital on health inequalities between migrant and local hypertensive patients is not surprising. Whether social capital mediates the relationship between socioeconomic status and health outcomes is still under debate. The term mediating refers to a third variable that underlies an observed relationship between two variables, i.e., indirect relation [30]. A study by Veenstra [22] found little evidence for the individual effects of social capital on SRH and no evidence for a mediating mechanism. However, the study by Lindstrom et al. [11] supported the idea that social capital is an important mediating link behind the socioeconomic differences in cardiovascular diseases. Although we did not observe that the lower levels of structural social capital for migrant hypertensive patients contributed greatly to health inequalities, these findings do not negate the importance of structural social capital as a potential contributor to the health of migrant hypertensive patients, since we did find that structural social capital was a predictor of SRH.

The limitations of the study should be addressed. First, the cross-sectional nature of the current study prevents us from establishing causality between registration status and social capital, as well as between social capital and health outcomes. Second, the generalizability of study findings was limited. The study was on-site based with the samples selected from health service users. In addition, we did not use random sampling method as there was an unavailability of exact sampling frame, although this systematic sampling method might replicate a random sampling method. Furthermore, cautions should be made when generalizing the current study findings to other cities. Third, although a set of socio-demographic factors were adjusted when calculating estimates, there is likely residual confounding due to omitted variables. Fourth, the questions used for the measurement of structural social capital may not completely capture the concepts we attempted to measure. Fifth, the information for structural social capital and SRH was self-reported and recall bias may have been introduced.

## Conclusions

Our study suggests that migrant hypertensive patients, in general, have lower structural social capital and poorer health outcomes than locals. Structural social capital contributes to health outcomes of hypertensive patients. Intervention strategies should emphasize the inclusion of migrant hypertensive patients in organizations or groups. Growing contacts that can provide support for migrant hypertensive patients should be an important goal of future strategies and policies. Our findings highlight the need for more research on mediating and moderating mechanisms in order to understand the relationship between registration status and health outcomes among hypertensive patients. Longitudinal research is needed to better elucidate causal inference between registration status, structural social capital and health outcomes across hypertensive patients.

## Consent for publication

All authors have read the manuscript and agreed to publish.

## Availability of data and material

The dataset supporting the results of this article is included within the article.
